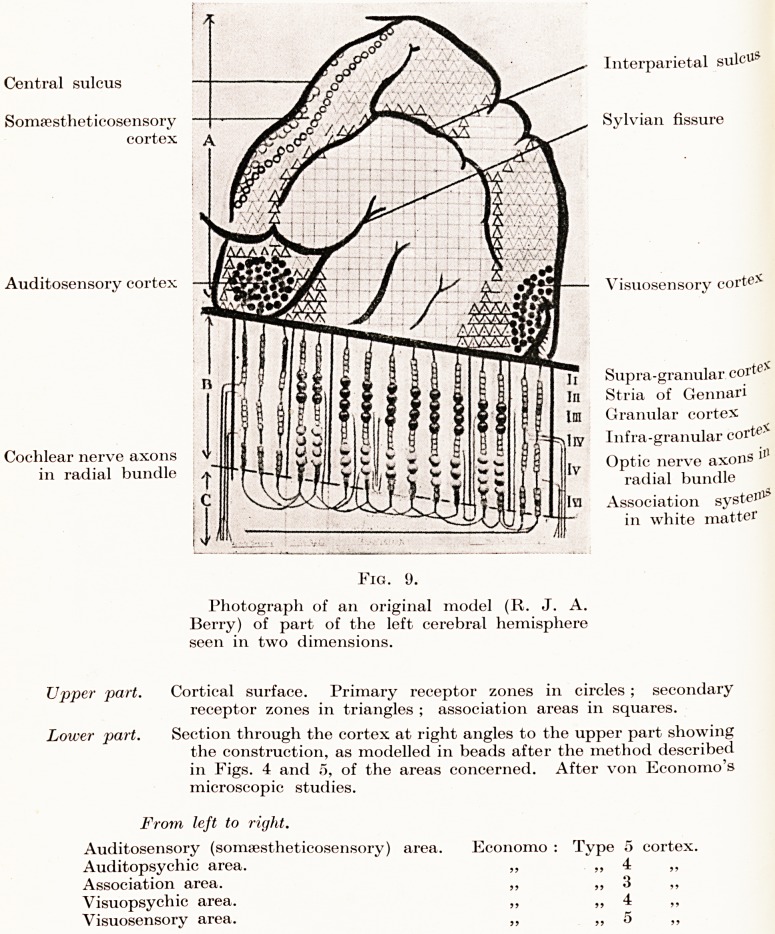# Cerebral Malformations and Their Clinical Consequences
*A demonstration given at the thirty-second Annual Meeting of the Association of Physicians of Great Britain and Ireland, held in Bristol on 2nd and 3rd June, 1938.


**Published:** 1938

**Authors:** R. J. A. Berry

**Affiliations:** Director of Medical Services at Stoke Park Colony


### CEREBRAL MALFORMATIONS AND THEIR CLINICAL CONSEQUENCES.*

BY

R. J. A. Berry, M.D., F.R.C.S., F.R.S.E.,

*Director of Medical Services at Stoke Park Colony.*

A series of brains chosen from the Stoke Park Collection formed a demonstration designed to show :—

1. The rate of growth of the normal human brain during the first few months and years of life.

2. The complete failure of the defective brain to achieve such normal growth, and

3. The additional occurrence amongst defectives of cerebral malformations of pre-natal origin and peculiarly gross kind.

To give the best ocular demonstration of the foregoing objectives six brains were exhibited in each group in three horizontal and parallel series, as follows :—

*A.* UPPER ROW. NORMALS.

| | 1 | 2 | 3 | 4 | 5 | 6 |
|---|---|---|---|---|---|---|
| Sex .. | Male. | Male. | Male. | Female. | Male. | Male. |
| Age .. | 2 days. | 2 mos. | 10 mos. | 2·6 yrs. | 4·0 yrs. | 6·0 yrs. |
| Product .. | 52 | 109 | 141 | 204 | 226 | 241 |
| Weight .. | 118 | 246 | 285 | 474 | 496 | 569 |

* A demonstration given at the thirty-second Annual Meeting of the Association of Physicians of Great Britain and Ireland, held in Bristol on 2nd and 3rd June, 1938.

*B.* MIDDLE ROW. MENTAL DEFECTIVES.

| | 1 | 2 | 3 | 4 | 5 | 6 |
|---|---|---|---|---|---|---|
| Sex .. | Male. | Female. | Male. | Male. | Female. | Female. |
| Age .. | 5·5 yrs. | 12·3 yrs. | 13·2 yrs. | 17·6 yrs. | 18·3 yrs. | 24·10 yrs. |
| Product .. | 148 | 132 | 186 | 175 | 107 | 206 |
| Weight .. | 319 | 310 | 404 | 396 | 242 | 443 |

*C.* LOWER ROW. CEREBRAL MALFORMATIONS OF PRENATAL ORIGIN. (MENTAL DEFECTIVES.)

| | 1 | 2 | 3 | 4 | 5 | 6 |
|---|---|---|---|---|---|---|
| Sex .. | Male. | Female. | Female. | Male. | Female. | Male. |
| Age .. | 7·1 yrs. | 13·5 yrs. | 33·8 yrs. | 4·4 yrs. | 12·7 yrs. | 7·2 yrs. |
| Product .. | 224 | 180 | 177 | 265 | 299 | 228 |
| Weight .. | 131 | 416 | 391 | 600 | 701 | 439 |

To establish a comparison between two such variants as the normal and defective brain it is of the first importance that the methods employed shall be strictly uniform throughout and shall be the same for both normals and defectives. When this investigation was first planned as one of the objectives of the then recently established Burden Mental Research Trust, Professor R. A. Fisher, of the Galton Eugenics Laboratory, was insistent that the defective brains should not be compared with the results obtained by others, but with entirely fresh normal material. Hence the necessity of establishing at Stoke Park a sufficiently large series of normal controls, eventually obtained from the Bristol Hospitals through the courtesy of their staffs. The two series—normal and defective—are therefore strictly comparable by any of the several methods employed—such as product or weight, *i.e.*

PLATE I

FIG. 1.

*Row A.*—Normals. *Row B.*—Mental Defectives. *Row C.*—Cerebral Malformations of Prenatal Origin. (Mental Defectives.)

the weight of the stripped and dried right cerebral hemisphere.

Anthropologists and biometrically-minded clinicians have long been accustomed to measure the length, breadth and height of the living head and/or skull, and have done so in accordance with the precise instructions of the British Association for the Advancement of Science.

To record the dimensions of these brains in the same way was therefore desirable, as the advantages of one standardized procedure for the measurement of living head, skull and brain are too great to need justification. This uniformity of method enables a direct clinical comparison to be instituted on the living subject between the size of his head during life and the probable size of his brain. The possibility of being able to prognosticate the size of a patient's brain during life may in the near future have an importance all its own.

*Product* as an index of size for living head, dried skull, or post-mortem brain is obtained by multiplying together the three dimensions of length, breadth and height, and taking the first three units of the answer, to the nearest whole number, as the product of size.

On examining the products of the six brains of the upper row normal series, it will be seen that at two days old the product is 52. Two months later it has doubled itself and is then 109. About the end of the first year of life the brain-product is about treble that at birth. Half way through the third year of life it is almost four times what it was at birth, and thereafter proceeds more slowly, so that at or about the sixth birthday the brain-product, as an index of size, is nearly five times that of the new-born infant.

The defectives of the middle row B present a very different picture. Here the defectives are arranged in chronological order of age from 5·5 years to 24·10 years, and a study of the brain-products shows that in no single instance is their brain-size more than that of a normal two-year-old infant. It is particularly instructive to compare each one of these defectives with the corresponding product figure for the normal infants of the upper row. Case No. 5 in Row B is unusually illuminating. She was one of six children of incestuous origin, the parents being father and daughter, five of them being particularly gross idiots. In the case of the brain illustrated, death occurred at the age of 18·3. Both brain-product and weight are less than those of the normal two-months-old male figured in Row A. She was a diplegic idiot, wet and dirty, unable to walk, stand, speak, feed, wash or dress herself.

It is equally instructive to examine the weights of the right cerebral hemispheres of the normals of the upper row and the defectives of the middle one. The same facts emerge. The average non-pathological defective, irrespective of his age, seldom surpasses the normal two or three-year-old infant. During its growth from birth to adult maturity the average brain increases in weight by roughly 1,000 grms., and this increment is largely, but not entirely, due to the myelinization of the very numerous cerebral axones of the white medullary centre. If, therefore, a defective brain is found to weigh less than a comparable normal —both having been weighed under strictly similar conditions—to be appreciably smaller in size, and to possess a corpus callosum (through which all medullated neopallial commissural axones must pass) smaller than the normal, it is a reasonable inference that that brain is deficient in its normal numbers of fully developed and properly functioning cortical neurones, and hence is incapable of achieving that type of mental reaction which distinguishes man from the animals.

It is these facts which give this comparison of defective and normal brains its importance. A study of the figures for the brain-size and weight as here given for the comparison of defective with normal is, in itself, a convincing explanation of why the defective is a defective.

In the third and lower row were exhibited six samples of the teratological monstrosities which pass for brains in some of the lower orders of defectives.

**No. 1** is an extraordinary case of cerebral agenesia, that is, absence of the greater part of the cerebral cortex. It occurred in a male aged 7.1 years. In view of the fact that the brain was a thin-walled bag of fluid, the patient's attitude foetal, and the reflexes those of a spinal animal, the most astonishing thing of all is that an unconscious existence could be maintained on a primitive brain-stem spinal cord mechanism for so long a period as seven years. In the first 120 defective brains of the Stoke Park collection cerebral agenesia occurred three times, of which the brain exhibited was the worst and most interesting example.

**No. 2** was a case of complete absence of the corpus callosum occurring in a girl who died at the age of 12.3 years, and who was regarded during life as being feeble-minded. Absence of the corpus callosum is a rare defect, but in the present series it was found completely absent three times, and partially so five times. Absence of the corpus callosum—partial or complete—is not in itself a sufficient cause of mental deficiency, but when, as in this instance, it is found in a brain which is too small for normal mental reactions, there is an adequate explanation of the girl's mental deficiency.

**No. 3** was an example of that very rare condition ossification of the dura mater with a terminal hæmorrhage into both lateral ventricles. It was found in a female aged at death 33.8 years. One of the results of the long-standing pressure from the bilateral ossified dura mater was an actual cortical destruction in the fronto-parietal operculum and adjacent superior temporal convolution. Professor T. B. Davie, who conducted the post-mortem examination, regarded the case as one of ossification in pachymeningitis hæmorrhagica interna due to congenital syphilis.

**No. 4** was from a male aged 4.4 years. His head during life was enormous and of a very abnormal shape—the auriculo-vertical height being 161 mm. On opening the skull, which was thin and with synostosed sutures, the subdural space was found to be filled, especially over the vertex, with a thick, glairy, gelatinous substance, which in some places was about a quarter of an inch in thickness.

**No. 5** was a case of megalencephaly occurring in a female aged 12.7. The total weight of the encephalon, unstripped, was 1,794 grms. That of the right cerebral hemisphere, stripped and dried, 701 grms. Whilst this brain was the heaviest in the defective series, still weightier examples of defective megalencephalic brains have been described, notably by the late Dr. Kinnier Wilson.

**No. 6** was one of the two examples occurring in the series of classical microgyria, and occurred in a male aged 7.2 years. Examples of partial microgyria were found in 16.7 per cent. of the defective brains.

The collection of brains from which these 18 examples were taken comprises 146 defective and a check series of 106 normal. The former have been obtained from consecutive post-mortem examinations at Stoke Park Colony conducted in the main by Professor T. B. Davie of the University of Bristol.

The importance of this collection to neurological science may be judged from the facts that the brains are entirely unselected, come from defectives of all grades and ages, both sexes, and from many different parts of England and Wales. All the brains are mounted, preserved and stored in such a way as to make them readily available to any other investigator

—the right cerebral hemisphere for naked-eye study and the left for microscopic. Whilst much has already been achieved with the material available, very much more remains to be done, and can be done given the necessary trained investigators.

A communication such as this, dealing with only one small part of the material available, is clearly not the most suitable for a full or detailed account of the many problems raised by the investigation. Some of this is already in the press and will shortly be published in the form of an Atlas which will include a full account of the social, mental, clinical and neurological histories of the patients during life.*

This demonstration raises certain outstanding points for discussion :—

(*a*) The relevancy of euthanasia to the speechless, helpless idiots and especially to those whose brains clearly reveal the impossibility of their being human in anything but outward form. There can be no doubt that any legislation for permitting euthanasia should include not only those normal individuals suffering from lingering, painful, incurable diseases, for whom it would be voluntary, but also a compulsory measure for the extirpation of those travesties of human life whose brains were exhibited.

(*b*) The ante-natal origin of these gross developmental and teratological errors of brain and nervous system appears to be incontrovertible. No one of these brains could be the outcome of any injury during birth to a brain which had developed normally up to the time of birth.

* *A Cerebral Atlas illustrating the differences between the brains of mentally defective and normal individuals, with a social, mental, and neurological record of* 120 *defectives during life.* With 430 photographs. By R. J. A. Berry. Oxford University Press. Ready about September.

Figs. 2, 3, 4 and 5.

Photographs of four original models by R. J. A. Berry, showing the structure of four of the five types of cerebral cortex recognized and described by von Economo :—

1. Agranular type. " Motor " cortex.
2. Frontal type of cortex.
3. Parietal type of cortex. " Associational " cortex.
4. Polar type of cortex.

These models are constructed from beads of different sizes and colours to represent nerve cells, and red and blue silks to represent effector and receptor axons.

Granular Golgi type II cells. White square beads.
Granular small pyramidal cells. Red square beads.
Medium pyramidal cells. Circular red beads.
Large pyramidal cells. Oval large red beads.
Ganglionic cells. Round blue beads.
Fusiform cells. Blue square beads.

In the left-hand column of each model are shown micro-photographs of von Economo's cortical types, the one illustrated being indicated by lines round it. Below this is the same author's map of the cerebral cortex showing the exact locality of each of the five types. To the right of the foregoing is a thin vertical band showing the corresponding division of the cortex, by G. A. Watson, into supra-granular, granular and infra-granular cortical laminæ. In the central column is the model of beads showing the exact cellular elements of each of von Economo's cortical types. The various cortical laminæ, as described by von Economo and Shaw Bolton, are lettered L and numbered in Roman numerals. In the right-hand column these several laminæ are also named in accord with nomenclature adopted by either von Economo or Shaw Bolton. Medullated axons are indicated running into, or out of, the white medullary centre.

### PLATE II

Fig. 2.

Fig. 3.

PLATE III

Fig. 4.

Fig. 5.

### PLATE IV

Bipolar receptor neurone

Single internuncial neurone

Effector neurone

FIG. 6.—The neuronic arc with single internuncial neuronic element.

Bipolar receptor neurone

Numerous Golgi type II or granular internuncial neurones

Effector neurone

FIG. 7.—A theoretically constructed neuronic arc with numerous granular internuncial neuronic elements.

Climbing fibres as receptor neurones

Cortical and basket cells as granulous internuncial neurones

Purkinje effector neurone

Moss fibres as receptor neurones

Stellate and granular cells as granulous internuncial neurones

FIG. 8.—The supra segmental neuronic arc as found in the cerebellum with very numerous internuncial neurons of granular type interposed between the incoming or receptor limbs of the arc, and the outgoing or effector limbs.

### PLATE V

Fig. 9.

Photograph of an original model (R. J. A. Berry) of part of the left cerebral hemisphere seen in two dimensions.

*Upper part.* Cortical surface. Primary receptor zones in circles; secondary receptor zones in triangles; association areas in squares.

*Lower part.* Section through the cortex at right angles to the upper part showing the construction, as modelled in beads after the method described in Figs. 4 and 5, of the areas concerned. After von Economo's microscopic studies.

*From left to right.*

| | | | | |
|---|---|---|---|---|
| Auditosensory (somæstheticosensory) area. | Economo : | Type | 5 | cortex. |
| Auditopsychic area. | ,, | ,, | 4 | ,, |
| Association area. | ,, | ,, | 3 | ,, |
| Visuopsychic area. | ,, | ,, | 4 | ,, |
| Visuosensory area. | ,, | ,, | 5 | ,, |

(*c*) The relationship of mental deficiency to criminal tendencies is more open to difference of opinion. One observer with first hand knowledge of one of the larger London prisons considers that feeble-mindedness is not common amongst the criminals in that prison. Others are inclined to the view more generally held that at least 50 per cent. of criminal offences are the work of the higher grades of feeble-minded or of the lower grades of dull normality.

(*d*) The utility of head measurement as a clinical aid, particularly amongst infants and young children, and the relative advantages of circumferential measurements over the more difficult ones of length, breadth and height, is a fruitful topic of discussion. Whatever individual preferences or prejudices may exist on craniometrical methods, the desirability of a closer contact between biometricians, clinicians and criminologists is universally admitted.

The use of various bead models of the spinal cord, cerebellum and cerebral cortex to explain some of the microscopic defects of the brain furnishes also a graphic method of demonstrating the dependence of some of the social problems of our times on these defects.*

When the teaching of neurological science can, with the aid of such models, be thus simplified and the subject be freed from some of the redundant nomenclature of its earlier days—and there is no reason why it should not be—the study of neurology and psychology will become more interesting, more instructive and more understandable. Then, too, it will achieve a greatly enhanced all-round utility.

* Figs. 2, 3, 4, 5, 6, 7, 8 and 9 are reproduced from models designed and invented by the author, and are included by kind permission of Mrs. R. G. Burden and Messrs. Macmillan & Co. Ltd.

It is most earnestly to be hoped that the study of the material at Stoke Park and demonstrations such as this will assist in bringing us back to those fundamentals of all mental science—anatomy, physiology and pathology—fundamentals from which psychology should never have departed.

## Figures and Tables

**Fig. 1. f1:**
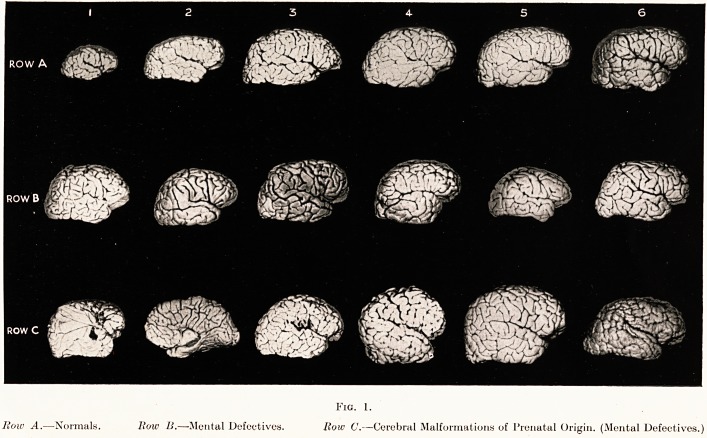


**Fig. 2. f2:**
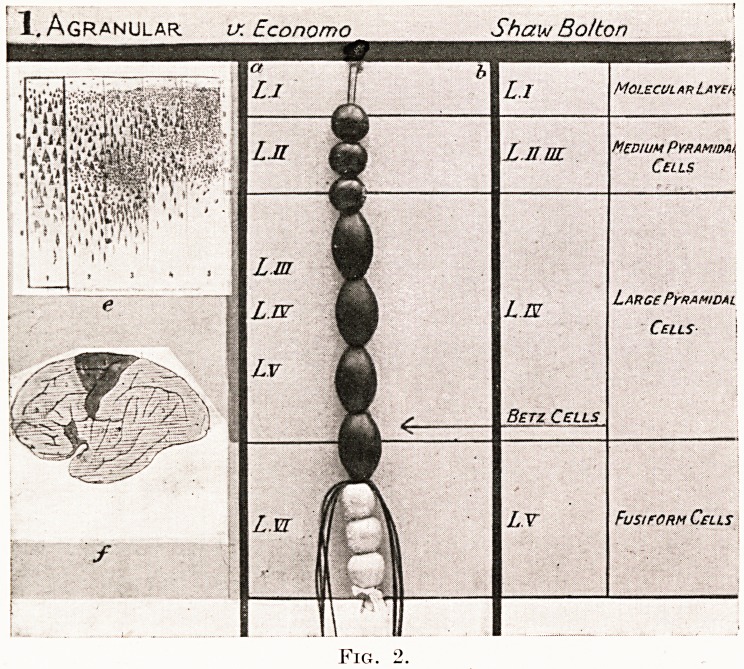


**Fig. 3. f3:**
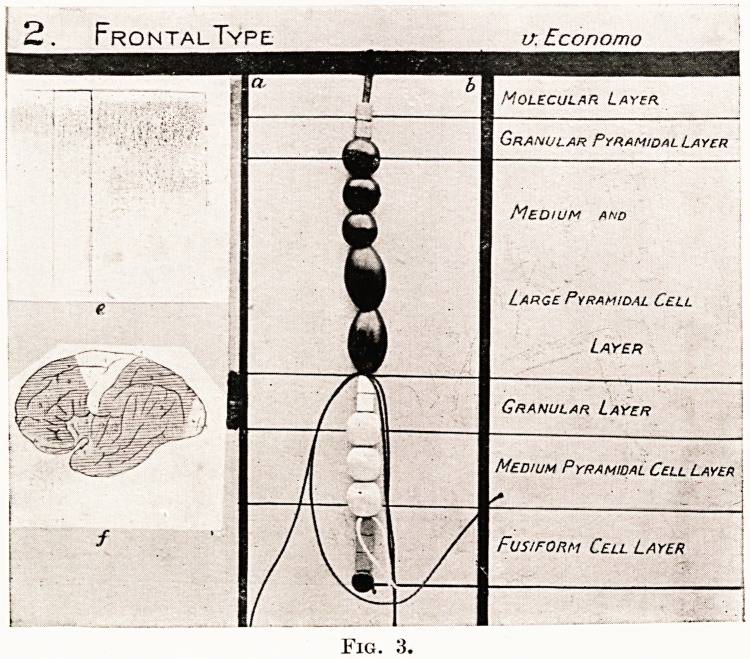


**Fig. 4. f4:**
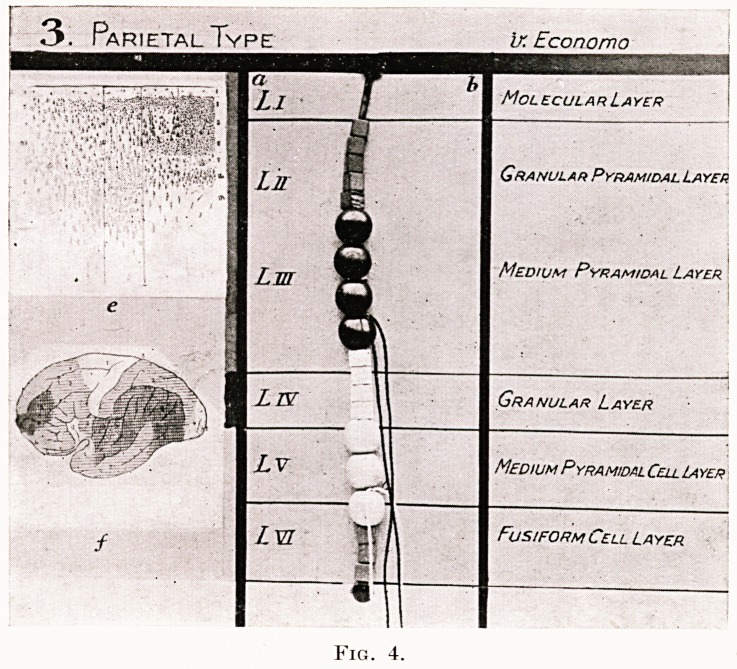


**Fig. 5. f5:**
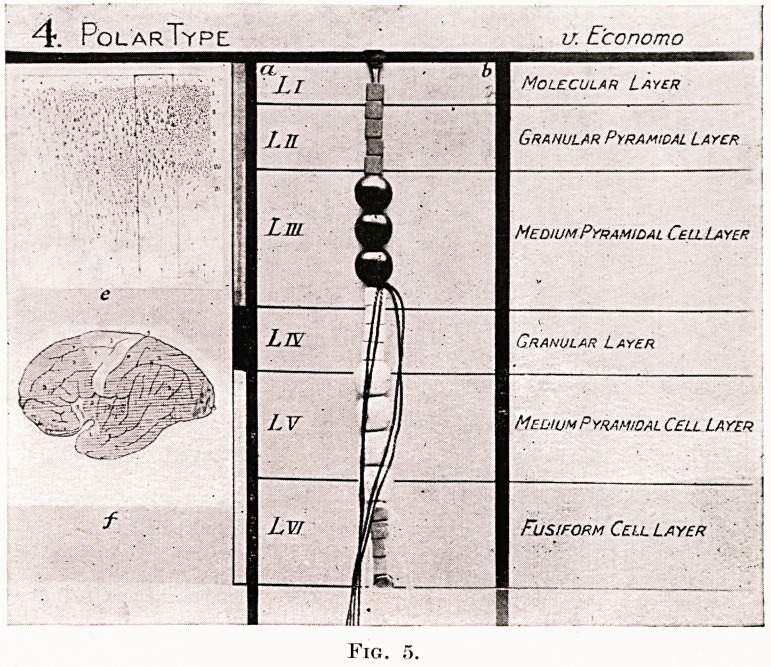


**Fig. 6. f6:**
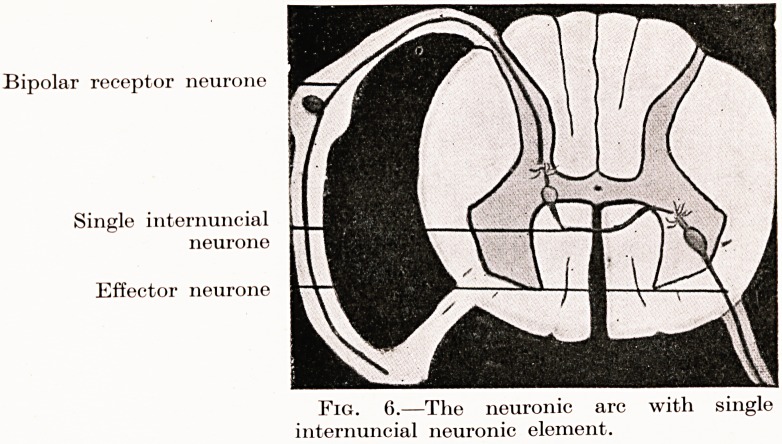


**Fig. 7. f7:**
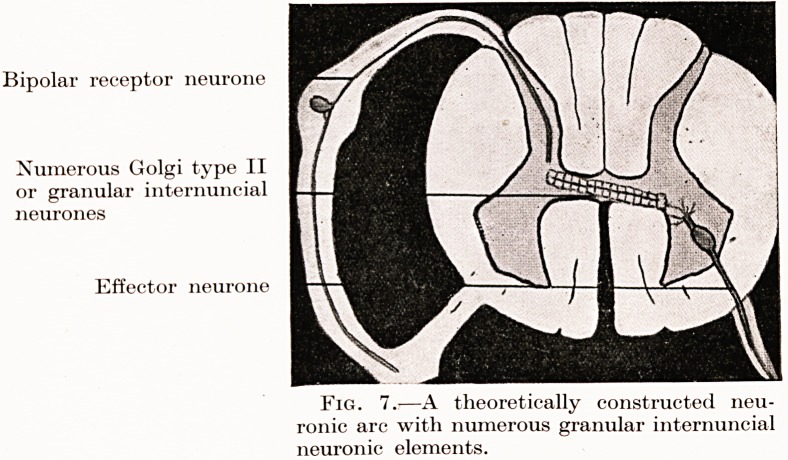


**Fig. 8. f8:**
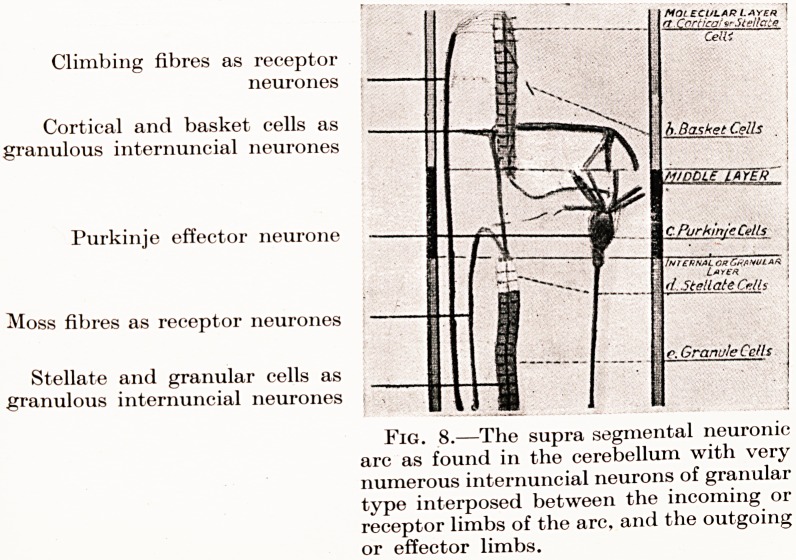


**Fig. 9. f9:**